# An early Cambrian greenhouse climate

**DOI:** 10.1126/sciadv.aar5690

**Published:** 2018-05-09

**Authors:** Thomas W. Hearing, Thomas H. P. Harvey, Mark Williams, Melanie J. Leng, Angela L. Lamb, Philip R. Wilby, Sarah E. Gabbott, Alexandre Pohl, Yannick Donnadieu

**Affiliations:** 1 School of Geography, Geology and the Environment, University of Leicester, University Road, Leicester LE1 7RH, UK.; 2 British Geological Survey, Keyworth, Nottingham NG12 5GG, UK.; 3 NERC Isotope Geoscience Facilities, British Geological Survey, Keyworth, Nottingham NG12 5GG, UK.; 4 Centre for Environmental Geochemistry, School of Biosciences, Sutton Bonington Campus, University of Nottingham, Loughborough LE12 5RD, UK.; 5 Aix Marseille Université, CNRS, IRD, Coll France, CEREGE, Aix-en-Provence, France.

## Abstract

The oceans of the early Cambrian (~541 to 509 million years ago) were the setting for a marked diversification of animal life. However, sea temperatures—a key component of the early Cambrian marine environment—remain unconstrained, in part because of a substantial time gap in the stable oxygen isotope (δ^18^O) record before the evolution of euconodonts. We show that previously overlooked sources of fossil biogenic phosphate have the potential to fill this gap. Pristine phosphatic microfossils from the Comley Limestones, UK, yield a robust δ^18^O signature, suggesting sea surface temperatures of 20° to 25°C at high southern paleolatitudes (~65°S to 70°S) between ~514 and 509 million years ago. These sea temperatures are consistent with the distribution of coeval evaporite and calcrete deposits, peak continental weathering rates, and also our climate model simulations for this interval. Our results support an early Cambrian greenhouse climate comparable to those of the late Mesozoic and early Cenozoic, offering a framework for exploring the interplay between biotic and environmental controls on Cambrian animal diversification.

## Teaser

An early Cambrian greenhouse climate constrained by combining oxygen isotope and climate modeling data approaches.

## INTRODUCTION

The oxygen isotope (δ^18^O) composition of fossil biominerals is one of the most widely used proxies for investigating ancient environments and is particularly useful as a deep time paleothermometer (*1*). The most widespread δ^18^O records come from analyses of calcium carbonate (*2*–*4*) and calcium phosphate (*5*–*7*) marine shelly fossils. However, although a near-continuous δ^18^O record exists back to the Early Ordovician epoch, 485 million years (Ma) ago (*1*, *6*), there is a substantial early Phanerozoic data gap that covers the interval of the Cambrian explosion, when the oldest identifiable fossils of most of the animal phyla appear in the rock record (*8*). This gap exists because phosphatic euconodonts with diagenetically robust hyaline crown tissues (*9*) are absent below uppermost Cambrian strata, whereas most calcareous fossils in these rocks have been demonstrably diagenetically altered (*1*, *4*). An alternative source of Cambrian oxygen isotope data is therefore needed to quantitatively assess the state of the early Cambrian climate, which has so far only been qualitatively interpreted as a greenhouse interval (*8*, *10*, *11*). We propose that “small shelly fossils” (SSFs) are a potential new source of δ^18^O data that could help constrain Cambrian marine environments.

SSFs comprise the skeletal elements of a diverse range of early biomineralizing animals that produced siliceous, calcareous, or phosphatic skeletons. Although many SSFs are preserved in secondary (diagenetic) phosphate (*12*), some are suggested to retain an original biophosphatic composition (*13*). To avoid sampling secondarily phosphatized specimens, we targeted fossils from the Comley Limestones, Shropshire, UK (fig. S1). These rocks are notable for yielding arthropods with limited (and very early) secondary phosphatization of soft anatomy but lack evidence for phosphatic replacement of original shell material (*14*). The Comley Limestones were deposited under normal marine conditions beneath the well-mixed waters of a shallow sea (*15*), approximately 65°S to 70°S on the peri-Gondwanan microcontinent Avalonia (*16*) between 514.45 ± 0.36 and 509.10 ± 0.22 Ma ago (*17*).

**Fig. 1:**
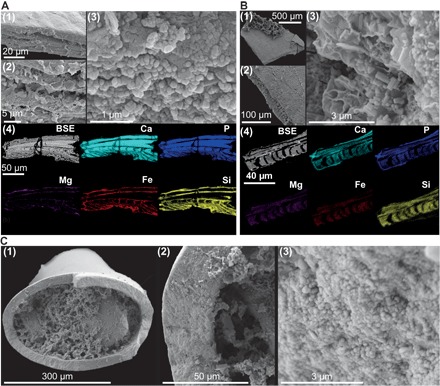
Preservation of linguliformean brachiopods and *Torellella* from the Comley Limestones. (**A**) SEM and EDX analyses of pristine brachiopods preserve alternating compact and porous phosphatic laminae [(1) and (2)]; compact laminae comprise densely packed phosphatic spherules (3). Diagenetically sensitive elements, particularly Fe and Mg, are restricted to porous laminae (4). BSE, backscattered electron image. (**B**) SEM and EDX analyses of altered brachiopods may preserve laminar microstructure, but compact laminae phosphate has recrystallized to micrometer-scale prismatic crystals (3). Diagenetically sensitive elements indicative of alteration are found throughout altered specimens (4). (**C**) Pristine *Torellella* specimens comprise densely packed phosphatic spherules a few tens of nanometers in diameter (3).

To ensure that isotope data recovered from the phosphatic SSFs reflect a Cambrian paleoenvironmental signal rather than later diagenetic conditions, we restricted our analyses to taxa known to have produced phosphatic skeletons based on phylogenetic and geological criteria (*13*). These included linguliformean brachiopods, which have well-known extant relatives, and the robust, thick-walled benthic tubular microfossil *Torellella*. We assembled taxon-specific samples for bulk isotope analysis comprising tens to a few hundred individual specimens and separated them according to their visual quality of preservation (fig. S2). Representative specimens from each bulk subset were subjected to a rigorous protocol to assess the preservation of biogenic phosphate. We could consistently distinguish subsets of pristine and altered SSFs using optical microscopy, confirmed by combining high-resolution scanning electron microscopy (SEM) and energy dispersive x-ray (EDX) spectroscopy to assess micro- and ultrastructural preservation and the distribution of diagenetically sensitive elements (see Materials and Methods). In particular, brachiopod specimens taken from pristine subsets were found to have compact laminae comprising phosphate spherules tens of nanometres in diameter, whereas the compact laminae of specimens taken from altered subsets were recrystallized as micrometer-scale phosphate prisms (Fig. 1). To help define a δ^18^O diagenetic gradient, we also analyzed an early diagenetic phosphate hardground, which likely formed at or close to the sediment-water interface, soon after deposition, from pore waters in communication with the overlying ocean (see Materials and Methods).

## RESULTS AND DISCUSSION

**Fig. 2:**
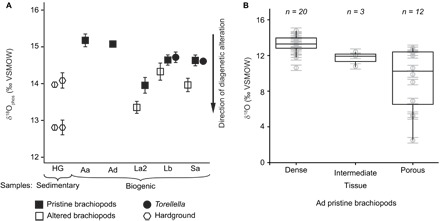
Early Cambrian δ^18^O_phos_ data from SSFs from the Comley Limestones. (**A**) In bulk analyses, altered subsets are isotopically lighter than the pristine counterparts from the same sample. Sample labels follow Table 1; error bars of 1 SD; δ^18^O_phos_ indicates trisilver phosphate analysis in which only phosphate oxygen isotopes were measured. (**B**) In situ (SIMS) data from linguliformean brachiopod specimens (sample Ad) show that porous laminae are consistently isotopically lighter than compact laminae. See data S1 and fig. S3. Box plots display the median and first and third quartiles, with the whiskers extending up to 1.5 times the interquartile range. All isotope data are reported relative to VSMOW.

Bulk oxygen isotope analyses (see Materials and Methods and data S1) of pristine phosphatic microfossils (five samples of linguliformean brachiopods and two of *Torellella*) yielded δ^18^O_phos_ values of +13.9 to +15.2 per mil (‰) Vienna standard mean ocean water (VSMOW). In contrast, three linguliformean brachiopod samples, identified a priori as being affected by diagenetic alteration, yielded lighter values of +13.3 to +14.3‰ (Fig. 2). Samples of sedimentary phosphate from an early diagenetic phosphatic hardground, in situ and as a rip-up clast, yielded still lighter δ^18^O_phos_ values ranging from +12.8 to +14.0‰.

In paired analyses of linguliformean brachiopods from the same sample, identified during our preanalysis screening as preserving either pristine or demonstrably altered phosphate, the pristine brachiopods consistently yielded heavier δ^18^O_phos_ than their diagenetically altered counterparts, by +0.3 to +1.0‰. The close correspondence of the δ^18^O_phos_ of diagenetically altered brachiopods (+13.3 to +14.3‰) to those of the phosphate hardground (+12.8 to +14.0‰) suggests that these altered SSFs may record early diagenetic conditions or at least have followed a similar diagenetic pathway as the hardground. In contrast, pristine *Torellella* samples yielded δ^18^O_phos_ values of 14.6 to 14.7‰, comparable to the pristine brachiopods (+13.9 to +15.2‰) and heavier values than those from the diagenetically altered brachiopods and the hardground samples. Most *Torellella* specimens were identified as pristine, and altered *Torellella* specimens were too scarce to assemble samples for bulk analysis (see Materials and Methods).

In situ secondary ion mass spectrometry (SIMS) analysis shows that oxygen isotopic preservation varies in the same manner as bulk elemental composition within individual brachiopod specimens (see Materials and Methods, fig. S3, and data S2). SIMS analysis consistently distinguishes between the δ^18^O values of compact and porous laminae, with compact laminae consistently heavier, by at least approximately 2‰, than porous laminae in the same specimen (Fig. 2). Bulk δ^18^O_phos_ values approximate to those of the compact laminae, likely because of the proportionately small contribution of the phosphate in the porous laminae to the total volume of shelly material analyzed (*18*, *19*). The systematic offset between the heavier values in the bulk δ^18^O_phos_ and lighter values from in situ SIMS analyses (Fig. 1) is likely due to the incorporation of all oxygen isotope species in a SIMS analysis spot [including the high proportion of structural carbonate in biogenic apatite (*19*)], compared with only phosphate-bound oxygen (PO_4_^3−^) measured by the trisilver bulk phosphate method. Because phosphate-oxygen temperature equations are based on trisilver δ^18^O_phos_ analyses (*7*, *20*–*22*), bulk δ^18^O_phos_ analyses are likely the most robust paleoenvironmental data from SSFs, with SIMS analyses providing critical information on intrasample variability and preservation.

### Isotopic composition of Cambrian seas

Selected SSFs from the Comley Limestones, screened to identify and exclude specimens unduly affected by diagenetic alteration, give δ^18^O_phos_ values that reflect shallow marine conditions on early Cambrian Avalonia. The δ^18^O_phos_ data incorporate signals from the oxygen isotopic composition of contemporaneous sea water (δ^18^O_sw_), the temperature of the water in which the animal lived, and biological fractionation (“vital effects”). The convergence of phosphate oxygen isotope temperature equations based on both marine invertebrate and nonmammalian marine vertebrate biominerals indicates that phosphate oxygen vital effects are small in comparison with analytical uncertainty (*21*). The impact of any vital effects in the phosphate oxygen isotope system is incorporated within the uncertainty of the empirically derived phosphate oxygen temperature (Eq. 1) (*22*)

$$T({}^{\circ}C)=(117.4\pm 9.5)-[(4.50\pm 0.43)*(\delta^{18}O_{\text{phos}}-\delta^{18}O_{\text{sw}})]$$ (1)

However, an estimate of δ^18^O_sw_ is still required. Local δ^18^O_sw_ is a function of the δ^18^O_sw_ of the global ocean average value, the local influence of freshwater input, and the regional precipitation-evaporation (P-E) balance (*23*). Secular variability of the global ocean δ^18^O reservoir, other than ice volume effects of ~1‰, is often disregarded in deep time paleoclimate studies (*5*, *6*) because of the potential buffering effect of balanced hot and cold hydrothermal alteration processes (*24*)—a view that has found some support from the emerging field of carbonate clumped isotopes (*25*–*28*). However, an invariant global ocean δ^18^O reservoir is at odds with the long-term carbonate, phosphate, and silica records, which all show comparable nonlinear secular trends in δ^18^O values (*2*, *4*, *5*, *29*, *30*). Over the Phanerozoic eon, this trend is thought to have shifted the δ^18^O value of the global ocean reservoir from approximately −6‰ in the early Cambrian to the heavier modern values of −1 to 0‰ (*2*, *4*, *30*). The δ^18^O_sw_ secular trend is further supported by similar magnitude trends derived from numerical modeling, with a substantial amount of this shift occurring in the early part of the Phanerozoic eon (*31*, *32*).

**Fig. 3:**
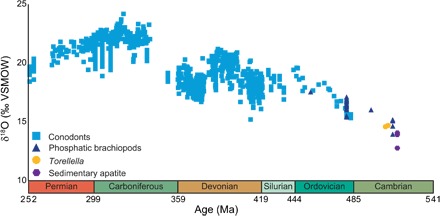
The Paleozoic phosphate δ^18^O record. Conodonts, blue squares; well-preserved linguliformean brachiopods, dark blue triangles; well-preserved *Torellella* samples, yellow circles; phosphate hardground data, purple hexagons). This trend is most pronounced in the earlier part of the Paleozoic. Data span a range of paleolatitudes and water depths. Our Cambrian data are essentially contemporaneous at approximately 513 Ma ago; for clarity, *Torellella* and phosphate hardground points have been shifted slightly on the age axis. All values are relative to VSMOW. See data S3.

Our new data support a secular trend in the global ocean δ^18^O reservoir and extend the biomineral isotope evidence for this trend back into the early Cambrian (Fig. 3 and data S3). We therefore suggest that paleoenvironmental δ^18^O studies in deep time should detrend raw data before making paleoenvironmental interpretations of temperature or ice volume change to account for this secular variation. To avoid the circularity of inferring both δ^18^O_sw_ and temperature from our data, we use preexisting whole-rock data (*4*) and geochemical modeling (*31*, *32*) to infer that global average early Cambrian δ^18^O_sw_ was approximately −6‰.

In addition to secular variability, we account for latitudinal P-E effects on local δ^18^O_sw_ in our temperature calculations—an important factor in paleoclimate research on more recent intervals but which is not usually considered in Paleozoic studies (*26*–*28*, *33*). Latitudinal P-E effects in modern oceans can be substantial, with surface δ^18^O_sw_ values ranging from −7.7 to +2.5‰ (*34*), although most of the global ocean is within ±1.5‰ of the global average (*23*, *34*, *35*). Isotope-enabled climate models for the early Cenozoic greenhouse climate suggest that the δ^18^O_sw_ distribution was similar to that of modern oceans, with perhaps slightly increased variability driven by an enhanced hydrological cycle (*36*, *37*). Given the well-connected position of Avalonia to the global ocean in the early Paleozoic (see Materials and Methods and fig. S1) and the range of δ^18^O_sw_ at comparable high southern latitudes in both the current icehouse and early Cenozoic greenhouse climate states (*34*–*37*), we use a conservative estimate of a −0.5‰ deviation from the Cambrian global average of −6‰ to give a δ^18^O_sw_ value of −6.5‰.

### Cambrian sea surface temperatures

**Fig. 4:**
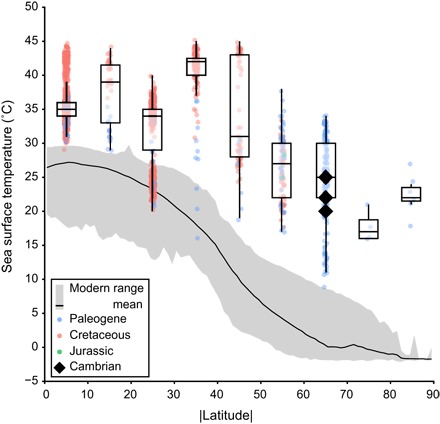
Cambrian isotopic SSTs in the context of Mesozoic and early Cenozoic greenhouse climate states. Cambrian data (black diamonds) from the δ^18^O_phos_ values of pristine SSF samples from the Comley Limestones. Data are plotted in 10° bins of the modulus of paleolatitude to illustrate latitudinal temperature variation irrespective of paleocontinental configuration. Plotted point data summarized in box plots displaying the median and first and third quartiles, with the whiskers extending up to 1.5 times the interquartile range. Cretaceous data, Cenomanian to Turonian; Paleogene data, Paleocene to Eocene. For references and literature data, see data S4. The modern latitudinal mean (black line) and range (gray envelope) data from the 2013 World Ocean Atlas 1° resolution data set (*60*).

Because the Comely Limestones were deposited in a shallow marine setting (*15*), we can interpret our isotope data as reflecting sea surface, rather than deep marine, conditions. Using the phosphate oxygen temperature (Eq. 1) (*22*) with a δ^18^O_sw_ value of −6.5‰, we reconstruct sea surface temperatures (SSTs) of 20° to 25°C for the Comley Limestones (Table 1). This is within the range of high-latitude temperatures of more recent greenhouse intervals in Earth’s history (Fig. 4 and data S4), such as those of the late Mesozoic and early Cenozoic (*38*–*41*).

**Table 1:** Bulk (trisilver phosphate) δ^18^O_phos_ data from the Comley Limestones and SSTs exploring the influence of different values for δ^18^O_sw_. Temperatures calculated from Eq. 1 (*22*). *T*_1_ δ^18^O_sw_ = −6.5‰, the most reasonable value; *T*_2_ δ^18^O_sw_ = −6‰, assumes no latitudinal P-E effects; *T*_3_ δ^18^O_sw_ = −1‰, the most commonly used Paleozoic value; *T*_4_ δ^18^O_sw_ = −8‰, incorporating the maximum likely latitudinal P-E effect of −2‰. Temperature uncertainty (2 SD *T*_1_) calculated from δ^18^O measurement SDs, assuming that δ^18^O_sw_ = −6.5‰. See data S1.

**Sample**	**Triplicate mean δ^18^O_phos_ (‰ VSMOW)**	**Triplicate 1 SD (‰ VSMOW)**	***T*_1_ (°C)**	***T*_2_ (°C)**	***T*_3_ (°C)**	***T*_4_ (°C)**	**2 SD *T*_1_ (°C)**
Pristine brachiopods							
Aa-Br-L	15.17	0.18	20	22	45	13	1.6
Ad-Br-L	15.06	0.03	20	23	45	14	0.3
La2-Br-L	13.93	0.22	25	28	50	19	2.0
Lb-Br-L	14.63	0.14	22	25	47	16	1.3
Sa-Br-L	14.62	0.15	22	25	47	16	1.3
Pristine* Torellella*							
Lb-To-To	14.70	0.14	22	24	47	15	1.3
Sa-To-To	14.59	0.07	22	25	47	16	0.6
Altered brachiopods							
La2-Br-D	13.33	0.17	28	30	53	21	1.5
Lb-Br-D	14.31	0.23	24	26	49	17	2.1
Sa-Br-D	13.94	0.18	25	28	50	19	1.7
Sedimentary phosphate							
HG-A	13.95	0.05	25	28	50	19	0.5
HG-A-DC	14.07	0.21	25	27	50	18	1.9
HG-B	12.77	0.09	31	33	55	24	0.8
HG-B-DC	12.78	0.20	31	33	55	24	1.8

Repeating our calculations assuming the ice-free Cenozoic value of −1‰ for δ^18^O_sw_ typically used in Paleozoic paleoclimate studies (*5*, *6*), rather than −6.5‰, our isotopic temperatures would shift from ~45° to 50°C (Table 1). This is unrealistic for high-latitude SSTs because they approach or exceed the lethal temperature limits for many marine animals, including brachiopods (*42*). We note that our δ^18^O_phos_ values are similar to those derived from well-preserved conodonts from the Tremadocian (Early Ordovician) of northern Gondwana (*5*) and Laurentia (*6*), which provide SST estimates of 40° to 44°C, assuming a δ^18^O_sw_ value of −1‰, albeit for lower latitudes. However, a recent paleoclimate modeling study encompassing the Early Ordovician epoch predicts much lower sea temperatures for the paleogeographic positions of these conodont δ^18^O data (*43*). Sea temperature estimates of 27° to 30°C, in much closer agreement with general circulation model (GCM) estimates (*43*), are obtained when a detrended Early Ordovician global ocean average δ^18^O_sw_ value of −4‰ (*32*) is used. Detrending the conodont δ^18^O_phos_ record to account for the observed δ^18^O secular trend (*2*, *4*, *29*) improves data-model comparison in early Paleozoic climate studies (*43*), providing further confidence in our δ^18^O_sw_ value.

### Paleoclimate implications

The characteristically warm high-latitude temperatures of greenhouse climate intervals, particularly noticeable in Mesozoic and Cenozoic paleotemperature proxy records (*38*–*41*), are consistent with our reconstructed SSTs (Fig. 4). Early Cambrian geological data generally support interpretations of a greenhouse climate state lacking permanent polar ice sheets, with evaporite and calcrete deposits spanning a wide paleolatitudinal range (*10*), the deposition of tropical soils (laterites) at high paleolatitudes (*44*), and a maximum of continental weathering rates over the past 900 Ma (*11*).

However, there may be some evidence for glaciation at high paleolatitudes (Avalonia) or even mid-paleolatitudes (Baltica) during the early Cambrian (*45*, *46*). Imprecise age constraints have hindered integration of these possible cold-climate deposits into the international stratigraphic framework, with some age estimates ranging from late Neoproterozoic to Early Ordovician. However, the likelihood is that these deposits (*45*, *46*) are of earliest (pretrilobitic) Cambrian or late Neoproterozoic age. Because our data from Cambrian Age 4 suggest climatic conditions that preclude even polar land ice at low altitude, it seems likely that any glacial activity was restricted to the earliest Cambrian or to short duration icehouse intervals.

**Fig. 5:**
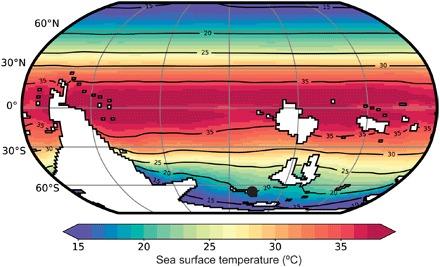
Early Cambrian mean annual SSTs, modeled by the FOAM GCM. The simulation was run under present-day orbital configurations with a CO_2_-equivalent greenhouse gas forcing of 32 PAL (see Materials and Methods and fig. S4). Black spot marks the position of our δ^18^O_phos_ data on Avalonia.

To further investigate the viability of our temperature estimates, we ran new GCM simulations of the early Cambrian climate (Fig. 5 and fig. S4) using the Fast Ocean Atmosphere Model (FOAM) (*47*)—a coupled ocean-atmosphere GCM that has recently been applied to interrogate other questions about early Paleozoic climates (*43*, *48*). The GCM simulations found good agreement with our new data for CO_2_-equivalent forcing of 32 times preindustrial atmospheric levels (PALs; 280 parts per million). This greenhouse gas forcing is in line with Cambrian *p*CO_2_ (partial pressure of CO_2_) estimates from GEOCARB suite of models (*49*). Both the data- and GCM-derived temperatures are comparable to late Mesozoic and early Cenozoic greenhouse conditions (Fig. 4) (*38*–*41*).

Overall, our new data provide the first quantitative constraints on early Cambrian climate, corroborating qualitative geological data and geochemical arguments that also support interpretations of this as a greenhouse world. Our new data fill an extensive time gap in the paleotemperature record of the beginning of the Phanerozoic eon and provide environmental context to a time when the animal-rich marine ecosystems of the Phanerozoic were first evolving. Using quantitative data to inform environmental and climate models will enable more rigorous interrogation of first-order hypotheses surrounding the ecological revolutions of the Cambrian Period, and these data can be recovered from globally widespread phosphatic Cambrian microfossils.

## MATERIALS AND METHODS

### Materials

#### The Comley Limestones

The Comley Limestones were deposited in a shallow sea (*15*) at ~65°S to 70°S on the peri-Gondwanan microcontinent Avalonia (*16*) between 514.45 ± 0.36 and 509.10 ± 0.22 Ma ago (fig. S1) (*17*). This fossiliferous and highly condensed limestone unit is less than 2 m thick, with five lithostratigraphic units (Ac_2_ to Ac_5_ and Ad) recognized on the basis of their petrographic and paleontological characteristics and separated by erosional disconformities that represent depositional hiatuses (*15*). Glauconite clasts and laminated iron-manganese nodules are common throughout the succession. The horizons are all more-or-less-sandy limestones, with detrital mineral abundance and faunal composition varying throughout the succession. The abundance of glauconite, laminated iron-manganese nodules, evidence for erosion and condensation, and the typically stenohaline trilobitic fauna indicates that there was no major freshwater (riverine) influence. There was deposition characteristic of normal marine conditions in the Welsh Basin throughout much of the Cambrian, Ordovician, and Silurian (*50*), and its peri-Gondwanan position close to the passive margin of the Iapetus Ocean during the Cambrian Period (*16*) suggests that it was well connected to the global ocean. The Comley Limestones are considered to have been deposited in an energetic shallow marine environment (*15*) that was unlikely to have been subjected to a seasonal thermocline.

#### Microfossil processing

Blocks of the Comley Limestones were macerated in buffered (10 to 15%) acetic acid using a standard extraction protocol, modified after Jeppsson *et al*. (*51*), that is not known to affect δ^18^O values (*9*). Heavy liquid separation, which is commonly used to concentrate microfossils within an acid residue but is known to affect δ^18^O_phos_ values (*9*), was not used. Acetic acid residues were collected between 1000- and 125-μm sieves and thoroughly rinsed with deionized water before being gently dried in an oven (*T* < 50°C). The residues were subsequently examined using a binocular microscope, and fossil specimens were picked out with a brush and deionized water. SSFs were assembled into 30-mg taxon-specific bulk samples, with each sample comprising several tens to a few hundred individual specimens.

Specimens were assembled into “pristine” and “altered” samples based on their appearance under optical microscopy. Pristine brachiopod specimens had a translucent light-brown appearance under reflected light, whereas altered specimens were opaque and appeared very dark brown to black (fig. S2). Specimens intermediate between these end-member states were excluded from further analysis. The vast majority of *Torellella* specimens were deemed pristine: light blue-gray in color, thick-walled, and with visible growth structures both within the tube walls and on the exterior surface. Although altered *Torellella* specimens exist (bleached white or black in color and with tube walls typically thin with no internal or external differentiation), no lithological sample yielded sufficient of these for bulk isotope analysis. The actual preservation state of samples was confirmed following the protocol outlined below.

#### Microfossil preservation

Because it was not possible to assess the preservation of every fossil in a bulk isotope sample, individual representative specimens were taken from bulk samples and examined to determine the typical preservation of each sample. Fractured surfaces of individual specimens were examined using high-magnification SEM to investigate the extent of recrystallization or overgrowth by diagenetic phosphate. Polished cross sections of individual specimens embedded in epoxy resin were examined using EDX spectroscopy to investigate the extent of chemical alteration and particularly the distribution of any such alteration within individual specimens.

The shells of modern linguliformean brachiopods comprised alternating dense layers of biophosphate (compact laminae) and organic-rich layers with much less biophosphate (porous laminae of various morphologies). The compact laminae, which constituted most of the biomineralized material, were composed of densely packed calcium phosphate spherules measuring from tens to a few hundred nanometers in diameter (*18*). We identified these submicrometer-scale phosphatic spherules in the compact laminae of specimens taken from pristine bulk samples (Fig. 1 and fig. S1). We also found these submicrometer-scale phosphatic spherules in specimens taken from pristine samples of the tubular SSF *Torellella* (Fig. 1). Specimens from samples identified as diagenetically altered showed recrystallization of these spherules, forming micrometer-scale phosphatic prisms (Fig. 1). These very small crystallite sizes, where microstructures are otherwise well preserved, may simply reflect solid-state recrystallization with no or minimal isotopic exchange because phosphate oxygen isotopes are known to be robust to low-temperature non–microbially mediated recrystallization (*52*). Secondary phosphatization was restricted to apatite overgrowths protruding into the porous laminae of specimens taken from samples of altered brachiopod samples. Overgrowths were also observed in some *Torellella* specimens, although where present, this was apparent under optical microscopy as bulges formed on the surfaces of these specimens wherever overgrowths had formed. *Torellella* specimens with these bulges were excluded from isotope analyses. In the most severely altered brachiopod specimens, all internal microstructure was lost, with the specimens being preserved as crude (blocky) outlines only.

Individual specimens from pristine and altered samples were embedded in epoxy resin and cured under pressure (approximately 2 bar) before being ground and polished with silicon carbide paper, diamond paste, and γ-alumina, before being thoroughly rinsed with ethanol and deionized water. EDX analyses of these specimens enabled us to map element distributions across different biological domains (compact and porous laminae). In brachiopod specimens taken from pristine samples, we found that elements indicative of alteration (most commonly, Fe, Mg, and Si) were restricted to porous laminae and were excluded from compact laminae (Fig. 1). In EDX analyses of specimens taken from altered samples, we found that these elements pervade the brachiopod compact laminae, where we had already observed prismatic phosphate recrystallization (Fig. 1). Having confirmed these preservation states with detailed SEM-EDX investigations, we determined that it was possible to separate pristine and altered specimens into bulk isotope samples using optical microscopy (fig. S2).

#### Phosphatic hardground

Sedimentary phosphate samples were also taken for bulk isotope analysis to provide a δ^18^O diagenetic gradient. These were acquired by microdrilling approximately 30 mg of powder from an irregular phosphatic hardground (HG-A) horizon in the lower part of the Comley Limestones and from a phosphatic pebble (HG-B) a few centimeters above the hardground. The in situ hardground is up to 3 cm deep, with an angular upper surface, and separates two distinct lithologies: an underlying trilobite-rich sandy limestone and an overlying glauconite-rich sandy limestone with few fossils. The overlying glauconite-rich unit also contains subrounded to angular phosphatic pebble-sized clasts. In places, the uppermost part of the hardground includes laminae of glauconite and quartz clasts.

Petrographic observations, including the lithological differences between the underlying and overlying units, the irregular upper surface, the preservation of calcareous fossils between the phosphate groundmass, and the fine glauconite and quartz laminae near the top of the horizon, suggested an early diagenetic origin of the phosphate, with occasional interruption of phosphate precipitation at the sediment-water interface and, ultimately, exposure of the hardground on the sea bed. Phosphate clasts were incorporated into the horizon above the hardground and were interpreted as deriving from the hardground during its exposure on the sea bed after its formation.

### Methods

#### Bulk isotope analyses

All δ^18^O values were reported with respect to VSMOW. Bulk isotope data were obtained from 30-mg fossil samples, with each sample comprising several tens of individual specimens, treated to solubilize PO_4_ anions and precipitated as silver phosphate [adapted after O’Neil *et al*. (*53*)] at the Natural Environment Research Council (NERC) Isotope Geoscience Facilities (NIGF). Samples of microfossils were crushed using a glass rod, cleaned in concentrated hydrogen peroxide for 24 hours to remove organic material, and subsequently evaporated to dryness. The samples were then dissolved in 2 M HNO_3_ and transferred to clean polypropylene test tubes. Each sample was then treated with 2 M KOH for neutralization and 2 M HF to remove calcium from the solution by precipitation of calcium fluoride. The samples were then centrifuged, and the supernatant was added to beakers containing ammoniacal silver nitrate solution and heated gently to precipitate silver phosphate. The silver phosphate was filtered, rinsed, dried, and weighed into silver capsules for analysis. Oxygen isotope measurements on each sample were analyzed in triplicate by continuous flow isotope ratio mass spectrometry (*54*). Analysis was via a high-temperature conversion elemental analyzer coupled to a Delta Plus XL isotope ratio mass spectrometer via a ConFlo III interface (Thermo Finnigan). The reference material B2207 (silver phosphate, Elemental Microanalysis) has an accepted value of 21.70‰, and the reproducibility of B2207 during this set of analyses was better than ±0.15 (1σ). All δ^18^O analyses were performed in triplicate, and the average SD of the triplicates was ±0.15‰.

#### Ion microprobe analyses

In situ SIMS analyses were conducted at the Edinburgh Ion Microprobe Facility (EIMF). For SIMS analysis, specimens were embedded under pressure (2 bar) in epoxy resin within 5 mm of the center of the block and around a central Durango apatite standard. Sample blocks were prepared using diamond grinding compounds, followed by diamond and alumina polishing compounds. Sample blocks were treated for 24 hours with H_2_O_2_ to remove organic matter from the embedded fossils. Surface reimpregnation, with minimal regrinding and polishing, was used to ensure a smooth and flat surface before gold coating for SIMS analysis.

SIMS analyses were made using the CAMECA IMS-1270 ion microprobe. A primary beam of Cs^+^ ions at ~5 nA was focused to a 30-μm-diameter spot on the sample block surface. Secondary ions were extracted at −10 kV, with ^16^O [~2 × 10^9^ counts/s (cps)] and ^18^O (~4 × 10^6^ cps) monitored simultaneously on dual Faraday cups (L’2 and H’2). Each analysis began with 50-s presputtering time, followed by automatic secondary beam and entrance slit centring, before data collection in two 10-cycle blocks. Each SIMS run began with 10 Durango analyses, followed by alternating analysis sets of five unknowns (samples) and five standards (Durango apatites), dropping to three standards when beam stability was good. Linear regressions were applied to each analysis run to correct for instrument drift. Mean external precision was derived from the SD of Durango analyses following linear regression corrections for long-term (session duration) drift. This value was reported for each unknown (sample) analysis and ranged from ±0.11 to 0.41‰. The Durango apatite standards were fragments of a larger crystal, supplied by EIMF, whose isotopic composition (δ^18^O_phos_, +8.7‰) was independently verified by trisilver phosphate analysis at NIGF before this project began.

#### Climate modeling

We used the three-dimensionally coupled ocean-atmosphere FOAM version 1.5 (*47*) that was widely applied to deep time paleoclimate studies (*43*, *48*, *55*, *56*). The atmospheric module is a parallelized version of the National Center for Atmospheric Research Community Climate Model 2 (CCM2), upgraded to include radiative and hydrologic physics from CCM3 version 3.2. We ran the atmospheric module with R15 spectral resolution (4.5° × 7.5°) and 18 vertical levels. The ocean module was the higher-resolution Ocean Model version 3, a 24-level *z*-coordinate ocean GCM giving 1.4° × 2.8° resolution on a regular longitude-latitude grid. The coupled model had no flux corrections, and its short turnaround time allowed millennial-scale integration.

We used the Cambrian continental configuration from BugPlates for 510 Ma ago (*57*). In the absence of land plants, the land surface was defined as a rocky desert (albedo, 0.24; modified by snow, if present). The solar luminosity was decreased by 4.3 % compared to its present value (1368 W m^−2^) (*58*), and orbital parameters were maintained constant to the present-day configuration. The *p*CO_2_ was fixed at 32 PALs (*59*), and concentrations of other greenhouse gases were kept to the present-day level so that the imposed radiative forcing must be considered a CO_2_ equivalent that may include some contribution of other greenhouse gases such as methane. The simulation was initialized using a warm ice-free ocean and a uniform salinity of 35‰. We integrated the model for 2000 years to reach deep-ocean equilibrium. During the last 100 years of the simulation, there was no apparent drift in the upper ocean and <0.01°C change in deep ocean (−3700 m) temperature. The last 50 years of the model run were used to build the climatology files used for analysis.

## Supplementary Material

http://advances.sciencemag.org/cgi/content/full/4/5/eaar5690/DC1

## SUPPLEMENTARY MATERIALS

Supplementary material for this article is available at http://advances.sciencemag.org/cgi/content/full/4/5/eaar5690/DC1 fig. S1. Paleogeographic and stratigraphic setting of the Comley Limestones (Avalonia, Cambrian Series 2). fig. S2. Examples of pristine and altered brachiopod and *Torellella* specimens. fig. S3. Box plots of ion microprobe (SIMS) data collected from pristine linguliformean brachiopods by tissue sampled. fig. S4. Global SST contour plots produced by early Cambrian FOAM GCM simulations for CO_2_-equivalent forcing of 32 PALs (see Materials and Methods). data S1. Triplicate trisilver phosphate isotope measurements. data S2. Processed ion microprobe (SIMS) data. data S3. Paleozoic phosphate δ^18^O data used to produce Fig. 3. data S4. Paleotemperature data used to produce Fig. 4. References (*61*–*124*)
