# Supplementary material for: An early Cambrian greenhouse climate
Source: Sci Adv. 2018 May 9;4(5):eaar5690. doi: 10.1126/sciadv.aar5690 (PMC5942912; doi:10.1126/sciadv.aar5690)
Supplement: http://advances.sciencemag.org/cgi/content/full/4/5/eaar5690/DC1 [file supp_4_5_eaar5690__index.html]

Science Advances | Science Advances

## Supplementary Materials

**This PDF file includes:**

- fig. S1. Paleogeographic and stratigraphic setting of the Comley Limestones (Avalonia, Cambrian Series 2).
- fig. S2. Examples of pristine and altered brachiopod and *Torellella* specimens.
- fig. S3. Box plots of ion microprobe (SIMS) data collected from pristine linguliformean brachiopods by tissue sampled.
- fig. S4. Global SST contour plots produced by early Cambrian FOAM GCM simulations for CO2-equivalent forcing of 32 PALs (see Materials and Methods).
- References (*61–124*)

Download PDF

**Other Supplementary Material for this manuscript includes the following:**

- data S1 (Microsoft Excel format). Triplicate trisilver phosphate isotope measurements.
- data S2 (Microsoft Excel format). Processed ion microprobe (SIMS) data.
- data S3 (Microsoft Excel format). Paleozoic phosphate δ18O data used to produce Fig. 3.
- data S4 (Microsoft Excel format). Paleotemperature data used to produce Fig. 4.

**Files in this Data Supplement:**

- Adobe PDF - aar5690\_SM.pdf
