# Supplementary material for: An early Cambrian greenhouse climate
Source: Sci Adv. 2018 May 9;4(5):eaar5690. doi: 10.1126/sciadv.aar5690 (PMC5942912; doi:10.1126/sciadv.aar5690)
Supplement: http://advances.sciencemag.org/cgi/content/full/4/5/eaar5690/DC1 [file aar5690_SM.pdf]

Science Advances

AAAS

advances.sciencemag.org/cgi/content/full/4/5/eaar5690/DC1

Supplementary Materials for

An early Cambrian greenhouse climate

Thomas W. Hearing, Thomas H. P. Harvey, Mark Williams, Melanie J. Leng, Angela L. Lamb, Philip R. Wilby, Sarah E. Gabbott, Alexandre Pohl, Yannick Donnadieu

Published 9 May 2018, *Sci. Adv.* **4**, eaar5690 (2018)
DOI: 10.1126/sciadv.aar5690

The PDF file includes:

- fig. S1. Paleogeographic and stratigraphic setting of the Comley Limestones (Avalonia, Cambrian Series 2).
- fig. S2. Examples of pristine and altered brachiopod and *Torellella* specimens.
- fig. S3. Box plots of ion microprobe (SIMS) data collected from pristine linguliformean brachiopods by tissue sampled.
- fig. S4. Global SST contour plots produced by early Cambrian FOAM GCM simulations for CO_2-equivalent forcing of 32 PALs (see Materials and Methods).
- References (*61–124*)

Other Supplementary Material for this manuscript includes the following:
(available at advances.sciencemag.org/cgi/content/full/4/5/eaar5690/DC1)

- data S1 (Microsoft Excel format). Triplicate trisilver phosphate isotope measurements.
- data S2 (Microsoft Excel format). Processed ion microprobe (SIMS) data.
- data S3 (Microsoft Excel format). Paleozoic phosphate $\delta^{18}O$ data used to produce Fig. 3.
- data S4 (Microsoft Excel format). Paleotemperature data used to produce Fig. 4.

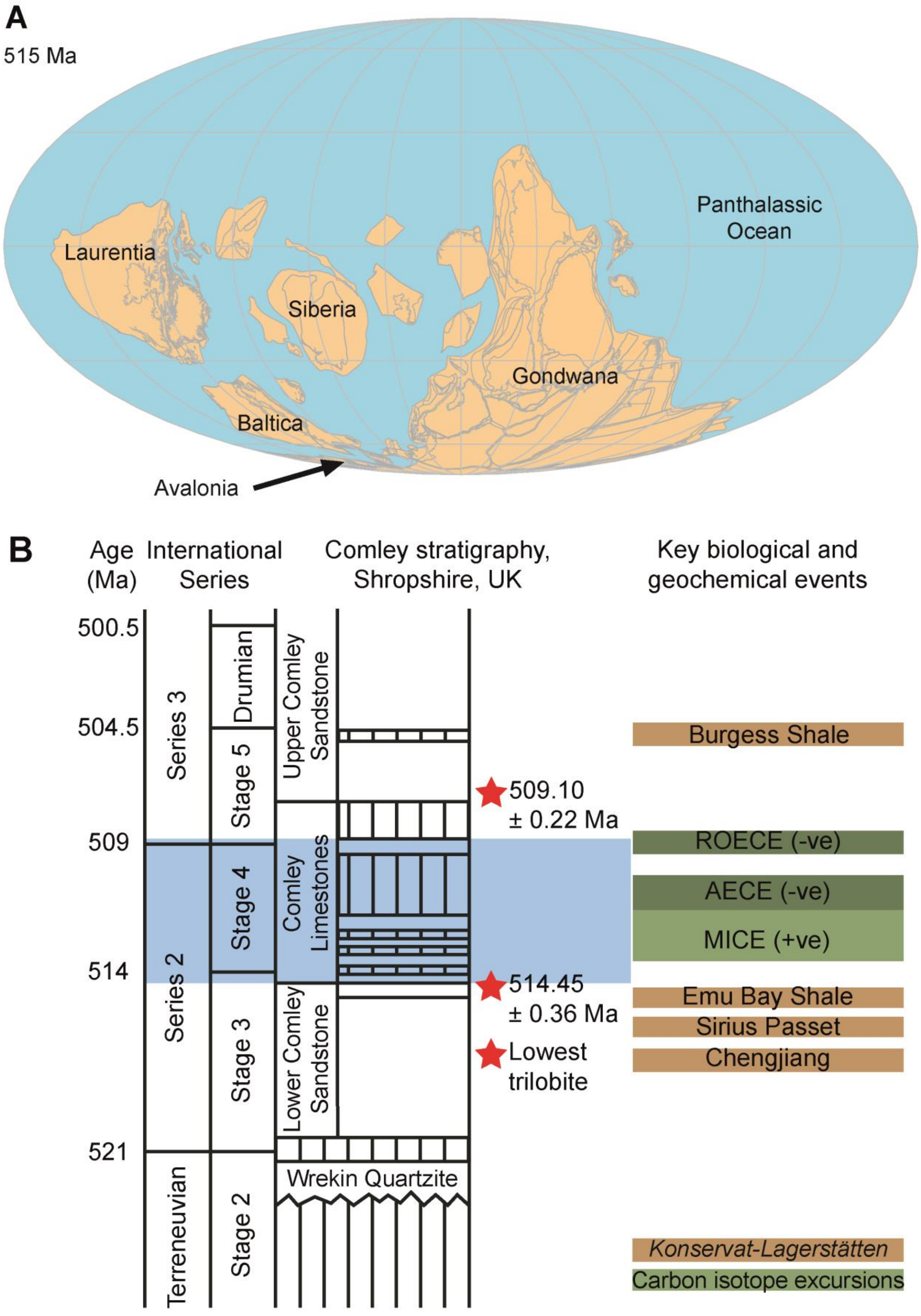
A
515 Ma
Laurentia
Siberia
Baltica
Avalonia
Gondwana
Panthalassic Ocean
B
Age (Ma)
International Series
Comley stratigraphy, Shropshire, UK
Key biological and geochemical events
500.5
504.5
509
514
521
Series 3
Series 2
Terreneuvian
Drumian
Stage 5
Stage 4
Stage 3
Stage 2
Upper Comley Sandstone
Comley Limestones
Lower Comley Sandstone
Wrekin Quartzite
509.10 ± 0.22 Ma
514.45 ± 0.36 Ma
Lowest trilobite
Burgess Shale
ROECE (-ve)
AECE (-ve)
MICE (+ve)
Emu Bay Shale
Sirius Passet
Chengjiang
Konservat-Lagerstätten
Carbon isotope excursions

fig. S1. Paleogeographic and stratigraphic setting of the Comley Limestones (Avalonia, Cambrian Series 2). Cambrian Series 2 paleogeographic (**A**) and stratigraphic setting (**B**) of the Avalonian Comley Limestones. (A) Paleogeographic setting of Avalonia and the major early Paleozoic continents at 515 Ma, after (*56*). (B) The Comley Limestones are chronologically constrained by radiometric dates (*17*) and can thus be integrated with the international chronostratigraphic framework. Green boxes mark prominent carbon isotope excursions (*60*); brown boxes indicate the deposition of the major Cambrian explosion *Konservat-Lagerstätten* (*61*). MICE: the Mingxinsi Carbon Isotope Excursion, associated with the Siberian archeocyathan radiation; AECE: the Archeocyathan Extinction Carbon isotope Excursion; ROECE: the Redlichiid and Olenellid trilobite Extinction Carbon isotope Excursion.

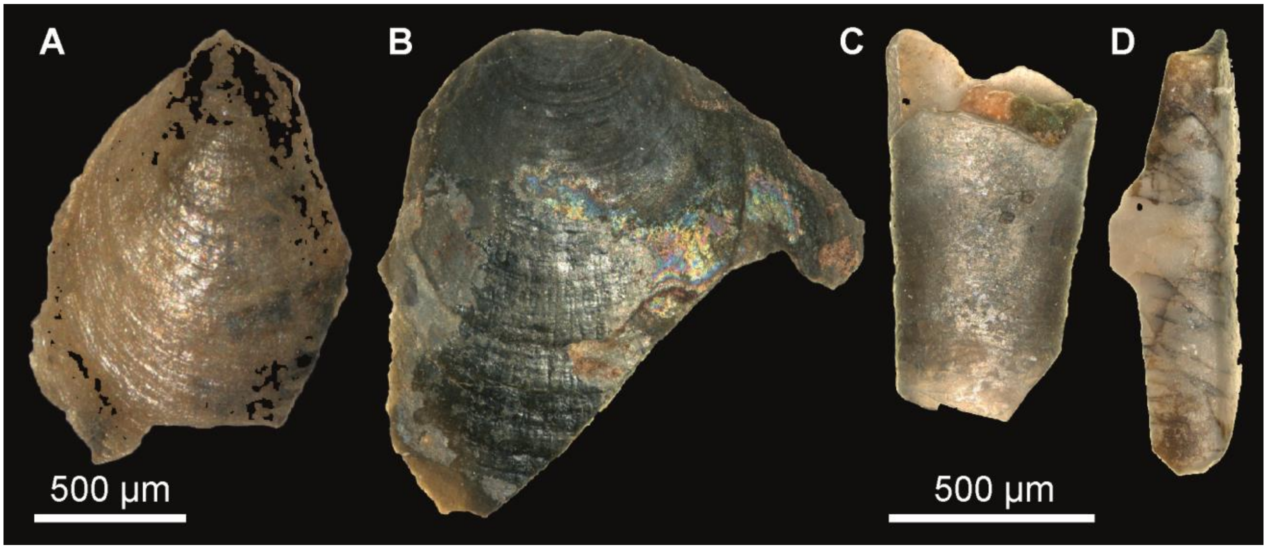

fig. S2. Examples of pristine and altered brachiopod and *Torellella* specimens. Examples of (**A**) pristine and (**B**) altered brachiopods, and (**C**) pristine and (**D**) altered *Torellella* specimens. Pristine brachiopods (A) are translucent green to light brown in colour, whereas altered brachiopods (B) are opaque dark brown to blue-black in colour and commonly have an oily sheen. Pristine *Torellella* specimens (C) have thick walls of light blue-grey phosphate with transverse lineations around the exterior surface and are unbroken by mineralised fractures. (D) Altered *Torellella* specimens typically have thinner phosphatic walls and fractures with iron and manganese oxide mineralisation. Images taken using an infinite focus microscope to allow in-focus imaging of specimens with high relief; the translucent quality of pristine brachiopods makes them hard to image and causes the patchy appearance of (A) at points where there was insufficient contrast to resolve a pixel.

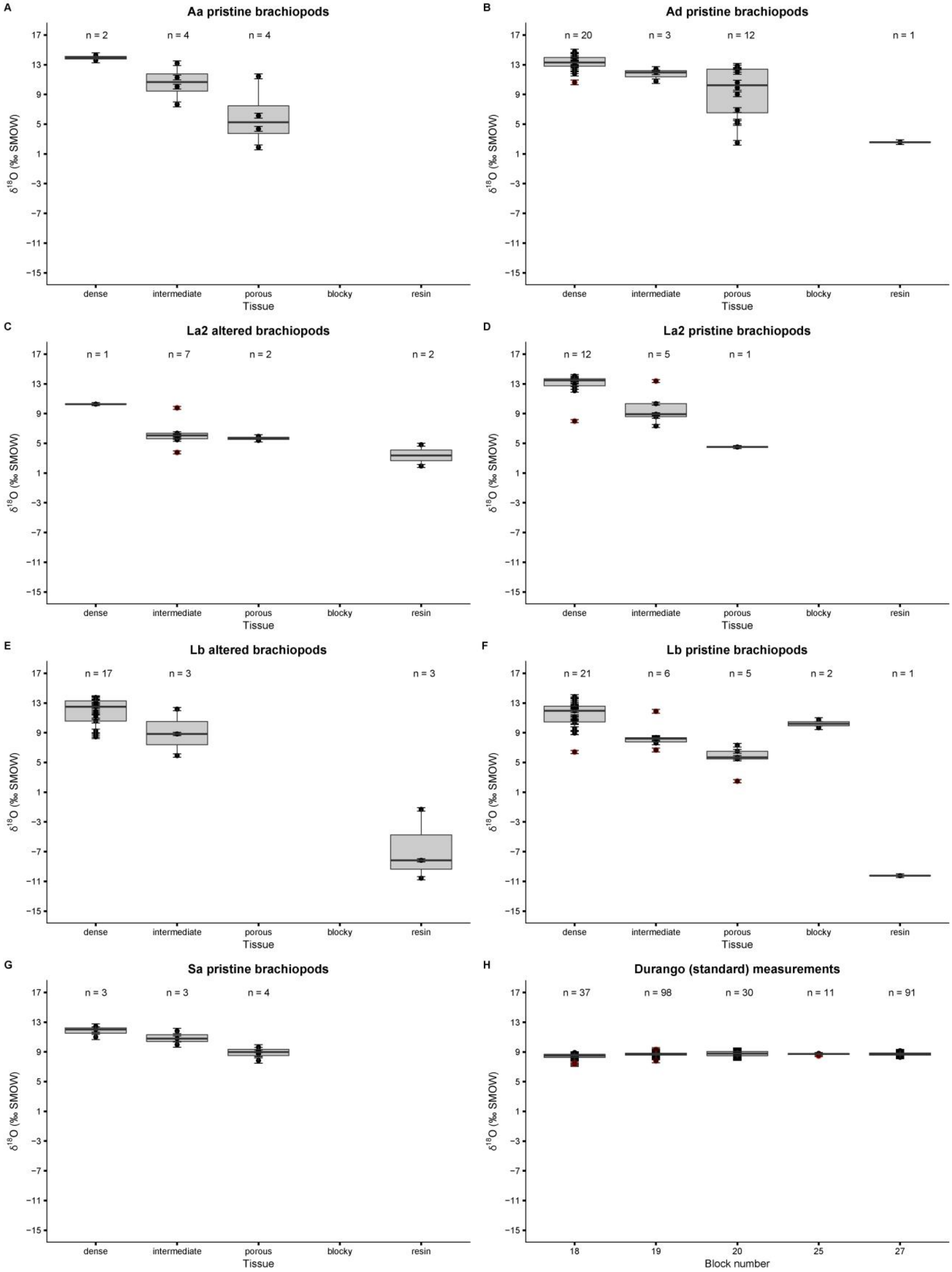
A
Aa pristine brachiopods
n = 2
n = 4
n = 4
B
Ad pristine brachiopods
n = 20
n = 3
n = 12
n = 1
C
La2 altered brachiopods
n = 1
n = 7
n = 2
n = 2
D
La2 pristine brachiopods
n = 12
n = 5
n = 1
E
Lb altered brachiopods
n = 17
n = 3
n = 3
F
Lb pristine brachiopods
n = 21
n = 6
n = 5
n = 2
n = 1
G
Sa pristine brachiopods
n = 3
n = 3
n = 4
H
Durango (standard) measurements
n = 37
n = 98
n = 30
n = 11
n = 91
δ18O (‰ SMOW)
17
13
9
5
1
−3
−7
−11
−15
dense
intermediate
porous
blocky
resin
Tissue
18
19
20
25
27
Block number

fig. S3. Box plots of ion microprobe (SIMS) data collected from pristine linguliformean brachiopods by tissue sampled. Compact laminae are consistently isotopically heavier than porous laminae in the same specimen and this is reflected in the averaged data. The porous laminae display a greater range of isotopic values than compact laminae, likely reflecting differences in the degree to which the porous laminae between individual specimens have been isotopically altered. X-axis labels: dense = compact laminae; porous = porous laminae; intermediate = combination of compact and porous laminae; blocky = no laminae visible on specimen, recrystallisation suspected; resin = analysis included both shell and resin. (A – G) Plot titles detail the sample ID (see Table 1); (H) boxplots of Durango measurements from each sample block. SIMS analysis incorporates $\delta^{18}O$ contributions from all oxygen phases present, whilst the bulk technique targets only the phosphate-bound oxygen. Biogenic apatite includes carbonate and hydroxyl oxygen, as well as phosphate within the crystal lattice. Our SIMS $\delta^{18}O$ data are consistently lighter than corresponding bulk $\delta^{18}O_{phos}$ data, but the offset is not systematic. We interpret this as reflecting variable chemical composition of linguliformean brachiopod shell chemistry, as well as potentially different degrees of diagenetic alteration of the carbonate oxygen isotope ratios between samples. Sample Lb has the smallest, but still clear, difference between pristine and altered brachiopods in $\delta^{18}O_{phos}$ data (Fig. 2), and this is reflected in the SIMS data (E, F) where the influence of structural carbonate $\delta^{18}O$ may be greater than the difference in $\delta^{18}O_{phos}$ data. This suggests that structural carbonate $\delta^{18}O$ may alter more readily than apatite $\delta^{18}O_{phos}$ (*63*, *64*). All boxplots display the median, and first and third quartiles, with the whiskers extending up to 1.5 times the interquartile range; the number above each box indicates the number of analysis points summarised in each boxplot.

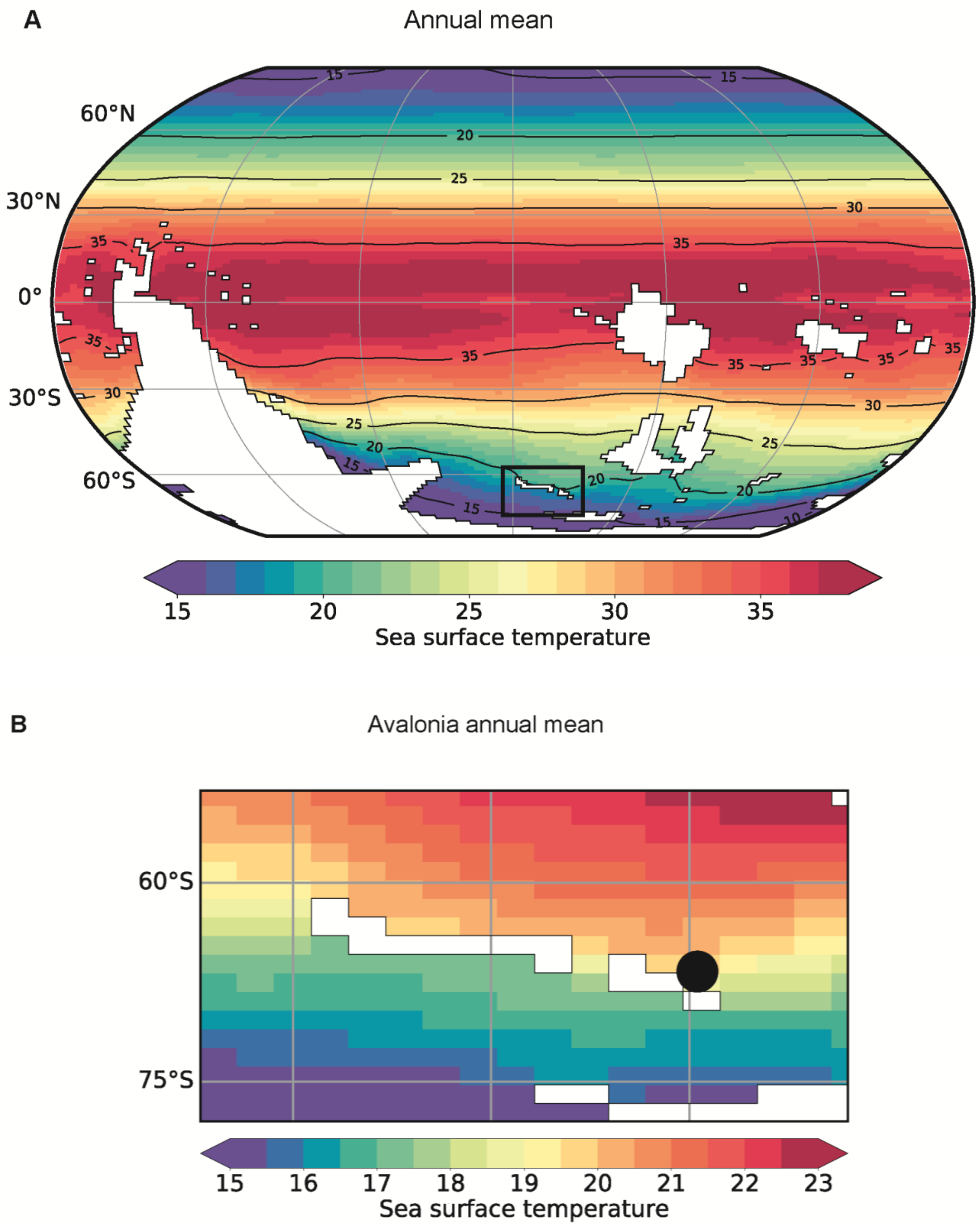

A
Annual mean
60°N
30°N
0°
30°S
60°S
15
20
25
30
35
10
Sea surface temperature
B
Avalonia annual mean
60°S
75°S
15
16
17
18
19
20
21
22
23
Sea surface temperature

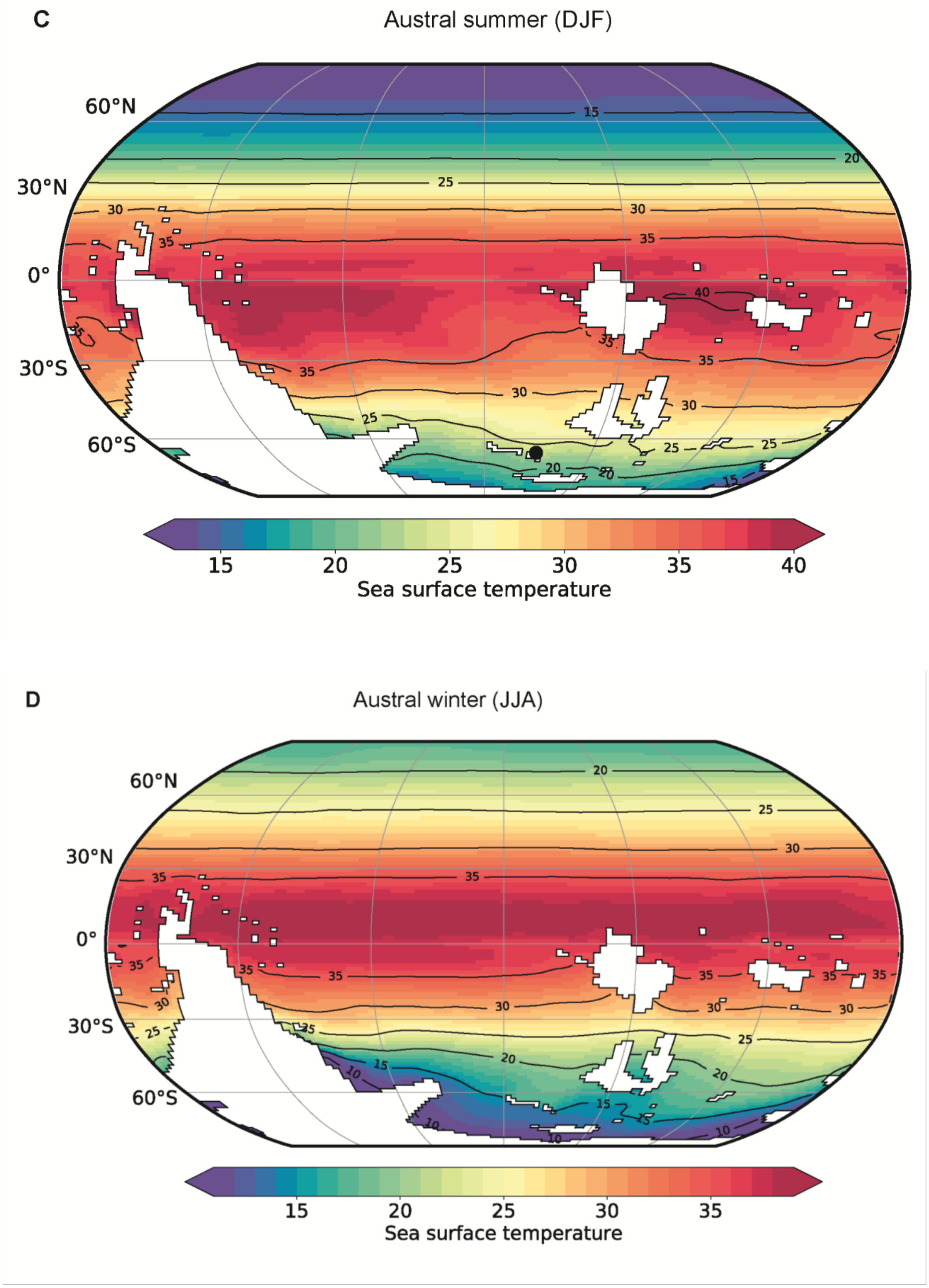
C
Austral summer (DJF)
60°N
30°N
0°
30°S
60°S
15
20
25
30
35
40
Sea surface temperature
D
Austral winter (JJA)
60°N
30°N
0°
30°S
60°S
15
20
25
30
35
Sea surface temperature

fig. S4. Global SST contour plots produced by early Cambrian FOAM GCM simulations for CO_2-equivalent forcing of 32 PALs (see Materials and Methods). (**A**) Mean annual SSTs; (**B**) austral summer SSTs, averaging December/January/February model temperatures; (**C**) austral winter SSTs, averaging June/July/August model temperatures. The position of Avalonia, and our data, is marked by a black spot. Basemap after (*57*) and others. Modelled Avalonia SSTs fluctuate between 15°C (winter average) to 24°C (summer average), comparable with our calculations from $\delta^{18}O_{phos}$ data.
